# Supplementary material for: Analysis of Three-Dimensional Cell Migration in Dopamine-Modified Poly(aspartic acid)-Based Hydrogels
Source: Gels. 2022 Jan 18;8(2):65. doi: 10.3390/gels8020065 (PMC8870902; doi:10.3390/gels8020065)
Supplement: Supplementary file 1 [file gels-08-00065-s001.zip › gels-1533910-supplementary.pdf]

gels

MDPI

Supplementary

Analysis of Three-Dimensional Cell Migration in Dopamine-Modified Poly(aspartic acid)-Based Hydrogels

David Juriga [1,*], Eszter Eva Kalman [2,3], Krisztina Toth [1,3], Dora Barczikai [1], David Szöllősi [4], Anna Földes [3], Gabor Varga [3], Miklos Zrinyi [1], Angela Jedlovszky-Hajdu [1,†] and Krisztina S. Nagy [1,3,*,†]

[1] Laboratory of Nanochemistry, Department of Biophysics and Radiation Biology, Semmelweis University, H-1089 Budapest, Hungary; toth.krisztina.105@gmail.com (K.T.); barczikai.dora@med.semmelweis-univ.hu (D.B.); mikloszrinyi@gmail.com (M.Z.); hajdu.angela@med.semmelweis-univ.hu (A.J.-H.)
[2] Department of Molecular Biology, Semmelweis University, H-1083 Budapest, Hungary; kalman.eszter@med.sememlweis-univ.hu
[3] Department of Oral Biology, Semmelweis University, H-1089 Budapest, Hungary; foldes.anna@dent.semmelweis-univ.hu (A.F.); varga.gabor@dent.semmelweis-univ.hu (G.V.)
[4] Department of Biophysics and Radiation Biology, Semmelweis University, H-1083 Budapest, Hungary; szollosi.sote@gmail.com
* Correspondence: juriga.david@med.semmelweis-univ.hu (D.J.); nagy.krisztina@dent.semmelweis-univ.hu (K.S.N.)
† These authors contributed equally to this work, therefore they both should be considered as last authors of this paper

Citation: Juriga, D.; Kalman, E.E.; Toth, K.; Barczikai, D.; Szöllősi, D.; Földes, A.; Varga, G.; Zrinyi, M.; Jedlovszky-Hajdu, A.; Nagy, K.S. Analysis of Three-Dimensional Cell Migration in Dopamine-Modified Poly(aspartic acid)-Based Hydrogels. *Gels* **2022**, *8*, 65. https://doi.org/10.3390/gels8020065

Academic Editor: Viorel-Puiu Paun

Received: 15 December 2021
Accepted: 14 January 2022
Published: 18 January 2022

Publisher's Note: MDPI stays neutral with regard to jurisdictional claims in published maps and institutional affiliations.

Copyright: © 2022 by the authors. Licensee MDPI, Basel, Switzerland. This article is an open access article distributed under the terms and conditions of the Creative Commons Attribution (CC BY) license (https://creativecommons.org/licenses/by/4.0/).

1. Preparation of Dopamine Modified Poly(succinimide)s

In the beginning of the synthesis, PSI was dissolved in dimethyl sulfoxide (DMSO) (VWR; 99.9%) to obtain a solution of 25 *w*/*w*%. Different amounts of dopamine-hydrochloride (Table S1) were dissolved in DMSO, and then dibutylamine (DBA) (Sigma Aldrich; 99.5%) was added to the solutions to adjust the pH of the reaction mixtures. Afterwards, the two types of solutions were mixed and stirred at 65 °C for 5 days. The PSI-DA polymers were precipitated in distilled water and dried at 45 °C for 2 days. The dopamine/succinimide molar ratio of the synthesized polymers were 1/40, 1/20 and 1/10.

Table S1. Applied amount of the chemicals in PSI-DA synthesis.

	PSI (g)	DA*HCl (mg)	DBA (mg)
1/40	1	48.7	31.7
1/20	1	97.5	62.5
1/10	1	195	125

2. Preparation of DA Containing PASP-Based Hydrogels

To prepare DA containing hydrogels, the PSI-DA polymers were dissolved in DMSO to obtain 25 *w*/*w*% solutions. As crosslinkers, diaminobutane (DAB) and cystamine (CYS) were used simultaneously to obtain crosslinks in every 10th monomer unit. DAB and CYS were dissolved in DMSO with 1/1 molar ratio and DBA was added to the reaction mixture to adjust the pH. Afterwards, the two solutions were mixed and loaded into a 0.75 mm-thick glass frame to form gel films. After 1 day, the gels were removed and dipped into extended amount of DMSO to remove all of the non-reacted molecules. Then the gels were treated with pH8 imidazole buffer (c = 250 mM) to hydrolyze the succinimide rings into aspartic acid and form hydrogels. Then, the hydrogels were immersed in 0.1 M dithiotreitol/pH8 buffer solution to cleave the disulphide bonds and form free thiol groups in the polymer matrices. For the cell experiments, the gels were washed several times with phosphate buffer saline (PBS) (pH = 7.5, c = 150 mM). For cell experiments, gel discs were cut with diameter of 6 mm for cell viability assays and with diameter of 10 mm for 2 photon microscopic investigations.

3. PSI Gel Films after Preparation

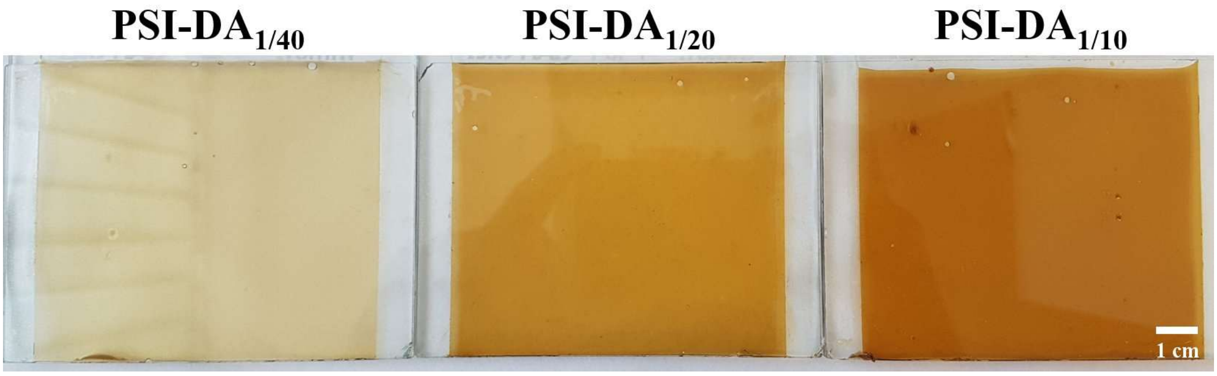

Figure S1. PSI-based gel films with different dopamine content.

4. UV-VIS Calibration Curve of DA

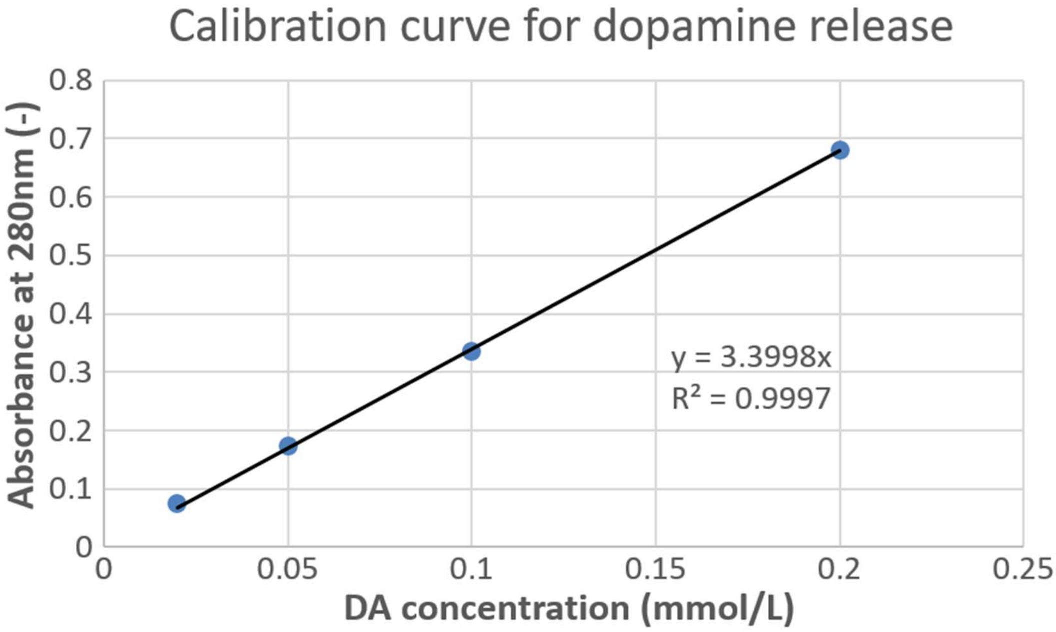

Figure S2. UV-VIS calibration curve of DA release measurement in ph8 buffer solution.

5. FTIR Spectrum Peaks List for Figure 2a

The peak at 3270 cm^{-1} is the asymmetric stretching of seconder –OH of the DA. The νC = O and νC-N stretching of amide group appeared at 1660 cm^{-1} and 1510 cm^{-1}, the signals of which also prove the successful modification of PSI. The peak intensity shows the same intensity waning than ν-OH. The characteristic peaks of imide group can be found in every spectrum at 1720 cm^{-1}. The measured spectra are similar to those reported in the literature [1,2].

6. Two-Photon Microscopic Analysis

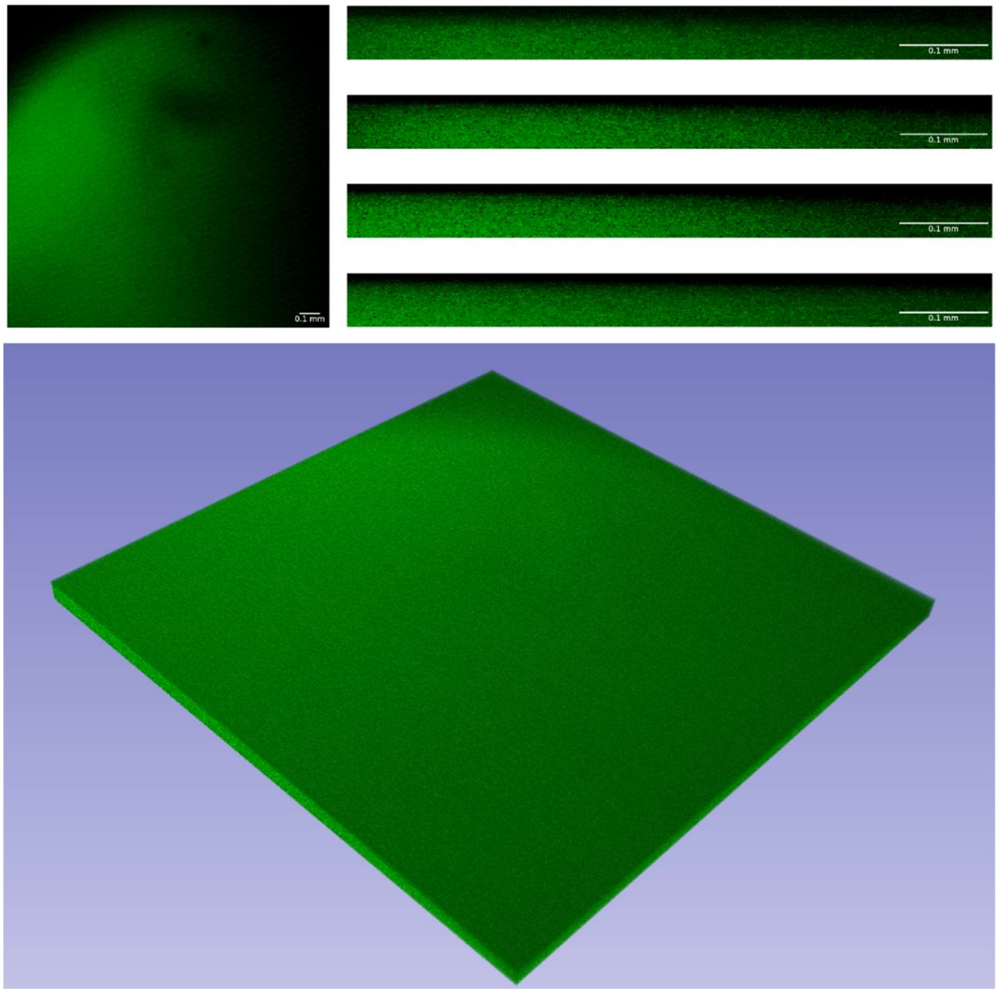

Figure S3. Upper view, cross-sectional view, and three-dimensional images were constructed from Z-stack measurements of a poly(aspartic acid)-based hydrogel. The hydrogel was treated with the same experimental method used in cell experiments. Scale bars indicate 0.1 mm.

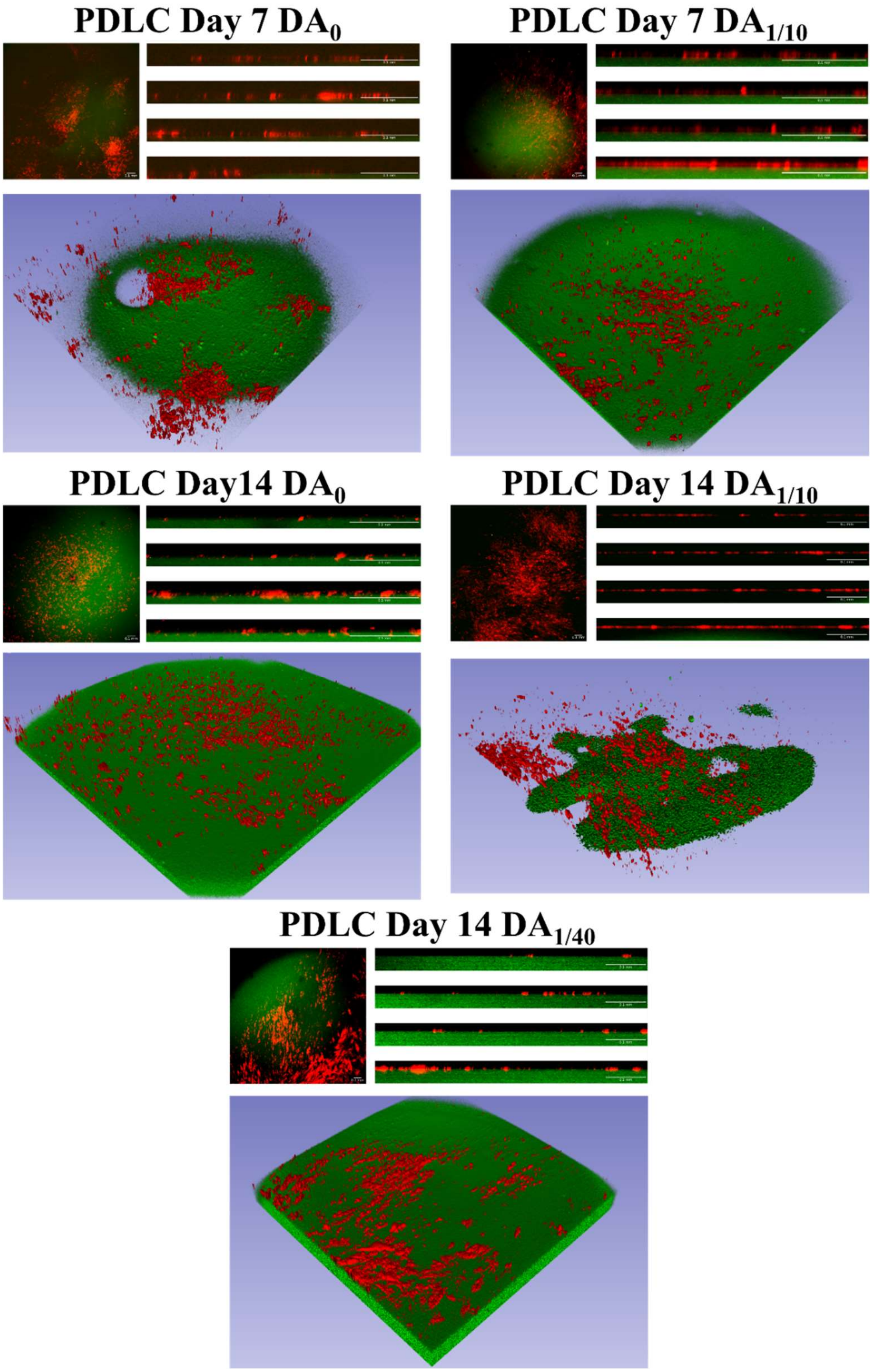

Figure S4. PDLC distribution (red) on the different hydrogel surfaces (green) on day 7 and 14. Upper view, cross-sectional view, and three-dimensional images were constructed from Z-stack measurements of the hydrogels. Scale bars indicate 0.1 mm.

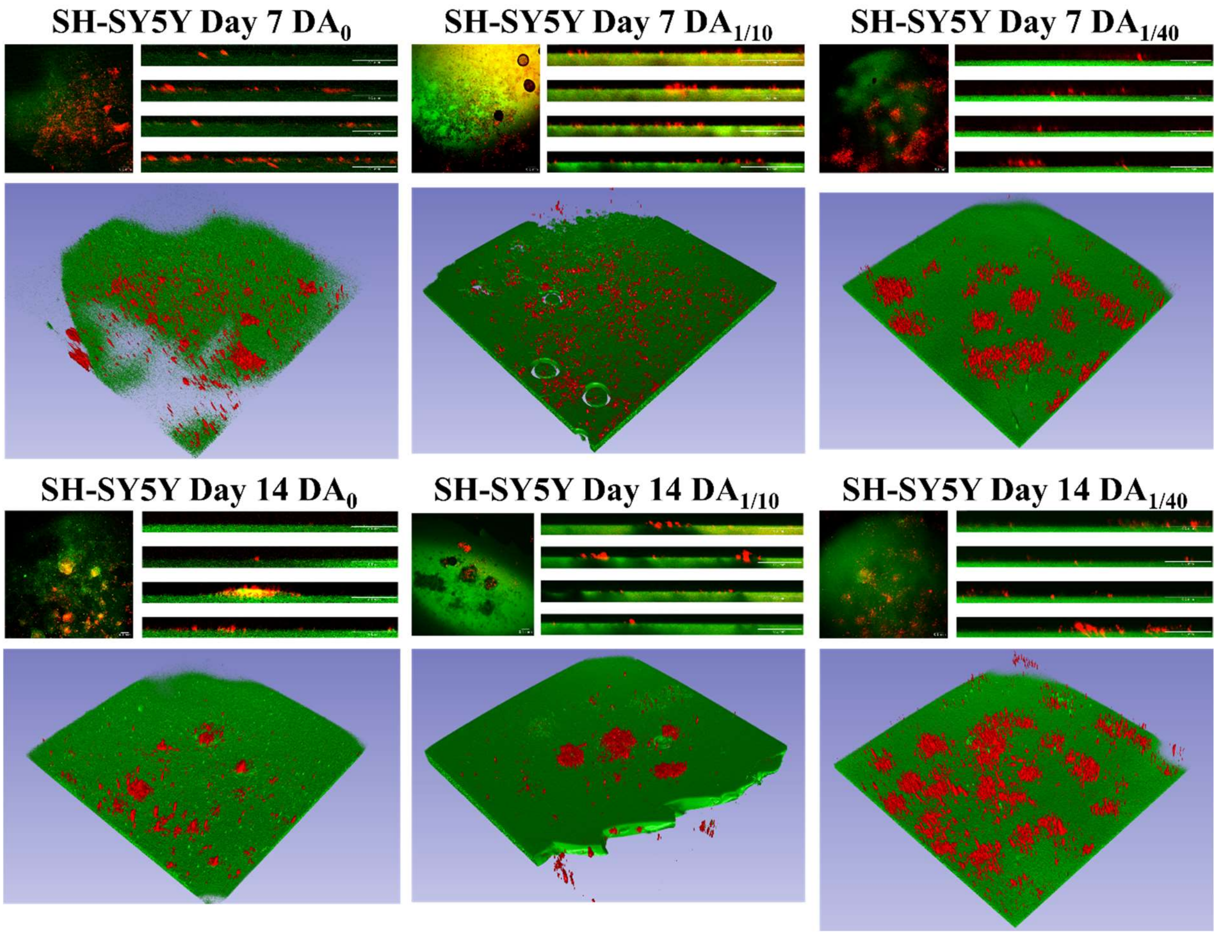

Figure S5. SH-SY5Y cell distribution (red) on the different hydrogel surfaces (green) on day 7 and 14. Upper view, cross-sectional view, and three-dimensional images were constructed from Z-stack measurements of the hydrogels. Scale bars indicate 0.1 mm.

References:

1. Juriga, D.; Laszlo, I.; Ludanyi, K.; Klebovich, I.; Chae, C.H.; Zrinyi, M. Kinetics of dopamine release from poly(aspartamide)-based prodrugs. *Acta Biomater.* **2018**, *76*, 225–238. https://doi.org/10.1016/j.actbio.2018.06.030.
2. Heo, S.B.; Jeon, Y.S.; Kim, Y.J.; Kim, S.H.; Kim, J.H. Bioinspired self-adhesive polymer for surface modification to improve antifouling property. *J. Coatings Technol. Res.* **2013**, *10*, 811–819. https://doi.org/10.1007/s11998-013-9528-9.
